# Detection of serum antibodies against *Leptospira* spp. in brown rats (*Rattus norvegicus*) from Grenada, West Indies

**DOI:** 10.14202/vetworld.2019.696-699

**Published:** 2019-05-23

**Authors:** Ravindra Nath Sharma, Katelyn Thille, Brianna Piechowski, Keshaw Tiwari

**Affiliations:** Department of Pathobiology, School of Veterinary Medicine, St. George’s University, Grenada, West Indies

**Keywords:** brown rats, enzyme-linked immunosorbent assay, Grenada, *Leptospira* spp

## Abstract

**Background and Aim:**

Leptospirosis is an emerging disease of animals and humans. Among rodents brown rats (*Rattus norvegicus*) are an important reservoir of bacteria *Leptospira*. There is a paucity of information on reservoirs of *Leptospira* in Grenada. This study was conducted to estimate the prevalence of antibodies against *Leptospira* spp. in brown rats in a densely human populated area of Grenada.

**Materials and Methods:**

Blood samples from 169 brown rats were collected and sera screened for antibodies against *Leptospira* spp. using enzyme-linked immunosorbent assay.

**Results:**

Among a total of 169 brown rats trapped in two parishes in Grenada, 77/169 (45.5%) were positive for *Leptospira* spp. antibodies. A significant difference in seropositive population of brown rats between two collection sites was observed. No differences were found between sex and age of seropositive rats.

**Conclusion:**

Due to the close contact of brown rats with humans in Grenada, rats should be considered a high-risk factor in transmission of *Leptospira* to humans. Appropriate preventive measures should be instituted to prevent the transmission of *Leptospira* infection to humans.

## Introduction

Leptospirosis is an emerging zoonosis in the world. Leptospirosis is caused by spirochetes of the genus *Leptospira* [1]. *Leptospira* spp. include pathogenic and non-pathogenic bacteria. Pathogenic *Leptospira* include eight species divided into 250 serotypes and 24 serogroups [2]. Leptospirosis is a disease of humans and animals. Humans can get infected from animal reservoirs. Among animals, rodents are important reservoirs of *Leptospira* [3,4].

Brown rats (*Rattus norvegicus*) are considered major natural reservoir of leptospirosis [5,6]. The most usual route of transmission is close contact with urine, within animal groups as well as humans. Mucosae (nose and eyes) and skin abrasions in contact with infected urine are the usual routes of infection. Occasional route of transmission is by ingestion of water and food contaminated with urine or tissues of infected animals. Bacteria entering the body circulate and finally localize in the kidneys. *Leptospira* colonizes in the proximal convoluted tubules of the kidney [7]. Monahan *et al*. [8] reported that persistent colonization in kidneys is due to immune privileged tissue of the kidneys. Chronically infected rats are asymptomatic, but they excrete *Leptospira* in the urine [9].

Leptospirosis has been reported from countries of South America and the Caribbean [10]. There are limited studies on leptospirosis in Grenada. Leptospirosis has been reported in cattle, pigs, sheep, goat, dogs, and chickens in Grenada [11-13]. With the exception of Keenan *et al*. [14] report, no published research is available on leptospirosis in brown rats from Grenada.

The aim of the current research was to estimate the serum antibodies against *Leptospira* spp. in brown rats from Grenada.

## Materials and Methods

### Ethical approval

The project (detection of zoonotic pathogens in brown rats (*R. norvegicus*) in Grenada) was approved by the Institutional Animal Care and Use Committee (IACUC ^#^16009-R) of the St. George’s University, Grenada.

### Study area

Grenada is the southernmost country in the Caribbean Sea with an area of 348.5 km^2^. The country with low hills, small trees, shrubs, and tropical climate is most suitable for rats. The country is comprised of six parishes: St. Patrick, St. Mark, St. Andrew, St. John, St. George, and St. David. St. David and St. George parishes, which have a higher human population compared to the other four parishes, were selected for the study.

### Collection of rats

A total of 169 rats were collected live from May 1 to July 14, 2017, using traps (45 cm l×15 cm w×15 cm h) with cheese and various local fruits as bait. Attempts were made to trap the rats from and near the residential buildings. Traps were placed 2 days/week in the evening and visited the morning of the next day. Traps with rats were covered with black cloth and transported to the necropsy laboratory of St. George’s University, School of Veterinary Medicine. Rats were anesthetized using 1-2% isoflurane in oxygen through portable vet anesthesia machine isoflurane vaporizer VET CE, manufacturer DRE (Avante Health Solutions Company, USA)

### Collection of samples and testing

The anesthetized rats were examined for their physical health and weighed. Gender was also recorded. Rats below 100 g were grouped as young and those over 100 g as adult, following the methodology used by Panti-May *et al*. [15]. Blood was collected from the heart through the thoracic wall and rats were exsanguinated this way. Sera were separated from the blood by centrifugation at 1500 g for 15 min at room temperature and stored at −80°C until tested. Enzyme-linked immunosorbent assay (ELISA) test for *Leptospira* antibodies on sera was performed using rat *Leptospira* IgG ELISA kit from MyBioSource, Inc., San Diego, CA 92195-3308 USA. The antigen in the ELISA kit was whole *Leptospira biflexa* organisms.

### Statistical analysis

Data were analyzed using a Chi-squared analysis and stratified by gender, age, and parish of rats in Microsoft Excel 2017 software. Statistical significance was set at p=0.05.

## Results

Antibodies to *Leptospira* spp. were found in 77/169 (45.5%) rats of 169 tested. St. George parish had a higher number of positive rats 43/76 (56.5%) compared to St. David, where 34/93 (36.5%) rats were seropositive. Male and female and young and adult rats had equal prevalence of antibodies (male 45.9% and female 43.9%; young 42.1% and adults 46.0%). There was no statistical significance between gender and age. The serological results of ELISA according to parish, gender, and age are presented in Table 1.

## Discussion

Diagnosis for leptospirosis is usually performed by serology: ELISA or microscopic agglutination tests (MAT) [4]. Serology is not useful in acute disease as antibodies develop in later stages of the disease. In recent years, PCR techniques have been used for the diagnosis of leptospirosis [4]. The detection of leptospires in tissue samples is based on culture, staining, and immunofluorescence techniques [7].

Previous researchers have reported variation in seropositivity of *Leptospira* in rats in different countries. Koma *et al*. [16] reported a 22% prevalence of anti-*Leptospira* antibodies by ELISA in *R. norvegicus* in Northern Vietnam. In 2010, a survey for anti-*Leptospira* antibodies in *R. norvegicus* in Brazil revealed 63% of rats seropositive [17]. They used immunofluorescence on kidney smears to identify *Leptospira*. An earlier study in Brazil [5] reported 68.1% positive *R. norvegicus* for *Leptospira* antibodies diagnosed by MAT. Sharon *et al*. [18] found 92% of rat serum samples positive for anti-*Leptospira* antibodies in the Philippines by MAT. Norway rats in Maryland, USA, were found positive (65.3%) for anti*-Leptospira* antibodies [19] diagnosed by ELISA. In Caribbean nations, including Grenada, studies on *Leptospira* were confined to livestock and humans; however, in Grenada, only one study made by Keenan *et al*. [14] reported the seroprevalence of *Leptospira* in *R. norvegicus*. They identified 24.5% of rats positive for *Leptospira* antibodies by ELISA and 7.1% positive by MAT. Compared to the previous study [14] in Grenada, this study found higher seropositivity (45.5%) in *R. norvegicus* for *Leptospira* antibodies by ELISA. The prevalence of antibodies for *Leptospira* spp. ranged from 22% to 92% in different countries. The variation in the prevalence of antibodies against *Leptospira* in different geographical location may be due to changes in climate and environmental conditions [20].

Comparison of the prevalence of antibodies in different studies is also complicated by sample size and laboratory methods [21]. The antibody estimation in serological diagnosis is dependent on the *Leptospira* spp. antigen present in ELISA kit. The antigen component of ELISA differs from one to another ELISA kit; however, in most products, the nature of antigen is not described. Most kits use crude whole cell lysate as antigens [22]. Same authors [22] evaluated the diagnostic utility of recombinant antigens, which contain portions of genes encoding the leptospiral outer membrane protein (LipL32, Ompl1, LipL36, LipL41, and Hsp58) in serodiagnosis of leptospirosis. Researchers recommended LipL32 had the highest sensitivities in the acute and convalescent phages of the leptospirosis. Other recombinant antigens gave insignificant reaction. In the present study, we used ELISA which contained whole *L. biflexa* organism. Since the details of *L. biflexa* organism are not available, it would be inappropriate to compare our serological results with previous researchers using different antigens.

In the present study, statistically significant differences in the prevalence of antibodies for *Leptospira* in brown rats were found between St. George and St. David parish. The climatic and environmental conditions being the same in both parishes, the difference is not fully understood. Future research involving a higher number of rats from all six parishes of the country may be more insightful regarding the distribution of antibodies of *Leptospira* in rat population of Grenada.

In the present study, no significant differences were found in the prevalence of antibodies between young and adult rats which are in accordance with Suepaul *et al*. [23] for many species of animals, except rats where juvenile were more susceptible. Contrary to our finding; the previous researchers [24,25,26] observed higher prevalence in older rats.

There was no difference in antibody prevalence between male and female rats. This finding is in concurrence with the previous researcher [27], whereas Easterbrook *et* *al*. [19] reported a higher prevalence of antibodies in female rats in the USA. In human patients from 14 Caribbean countries, males had a higher frequency of seropositivity compared to females. [28]. The difference between genders is not well explained since both sexes are equally exposed to *Leptospira* infection.

## Conclusion

The high prevalence of antibodies found in brown rats for *Leptospira* spp. in Grenada should be of great concern. In Grenada, cases of *Leptospira* infections in humans have been reported [28]. Due to the close contact of brown rats with humans in Grenada, rats should be considered a high-risk factor in transmission of *Leptospira* to humans. Preventive measures should be directed toward minimizing the exposure of rats with humans and possible reduction in Grenada’s rat population.

## Authors’ Contributions

RNS planned and supervised the research and manuscript writing; KeT trapping of rats, collection of blood, helping in ELISA, statistical analysis of results, and review of the manuscript; KaT performed ELISA and edited manuscript; BP collection of blood, and performance of ELISA. All authors read and approved the final manuscript.
